# Supplementary material for: Investigating the Role of Heparanase in Breast Cancer Development Utilising the MMTV-PyMT Murine Model of Mammary Carcinoma
Source: Cancers (Basel). 2023 Jun 5;15(11):3062. doi: 10.3390/cancers15113062 (PMC10253202; doi:10.3390/cancers15113062)

Supplementary Materials

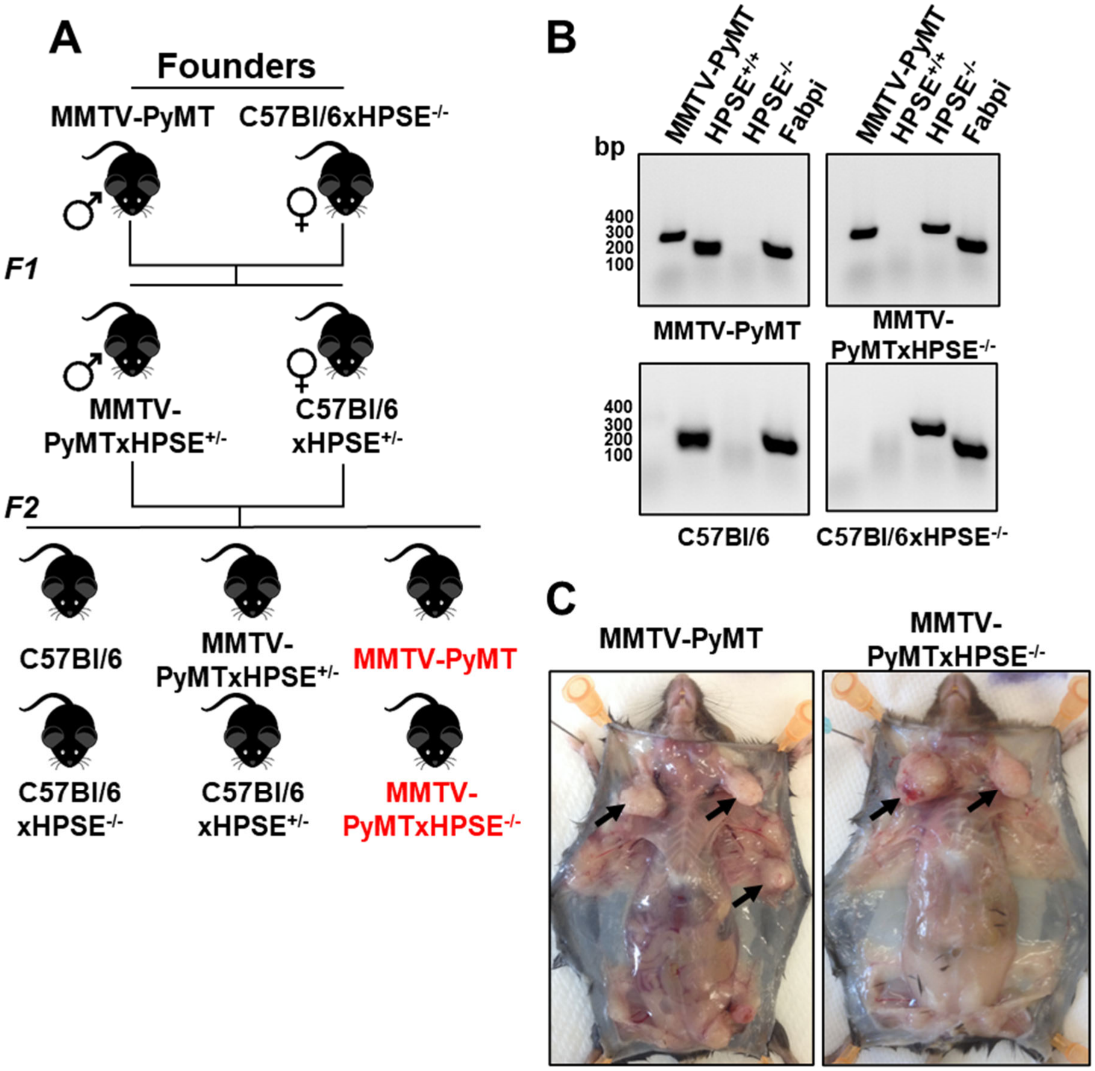

Supplementary Figure S1. The generation of MMTV-PyMTxHPSE$^{-/-}$ mice

(A) The schematic of the breeding strategy used to generate MMTV-PyMTxHPSE$^{-/-}$ mice. The genetic backgrounds of animals resulting from the *F2* generation that were used in subsequent *in vivo* studies are indicated in red. **(B)** Genotypes of MMTV-PyMT, MMTV-PyMTxHPSE$^{-/-}$, C57Bl/6 and C57Bl/6xHPSE$^{-/-}$ animals were confirmed by PCR. **(C)** Representative photographs of spontaneous mammary tumour-bearing female MMTV-PyMT and MMTV-PyMTxHPSE$^{-/-}$ mice at 20-weeks of age, showing visible mammary tumours (arrows).

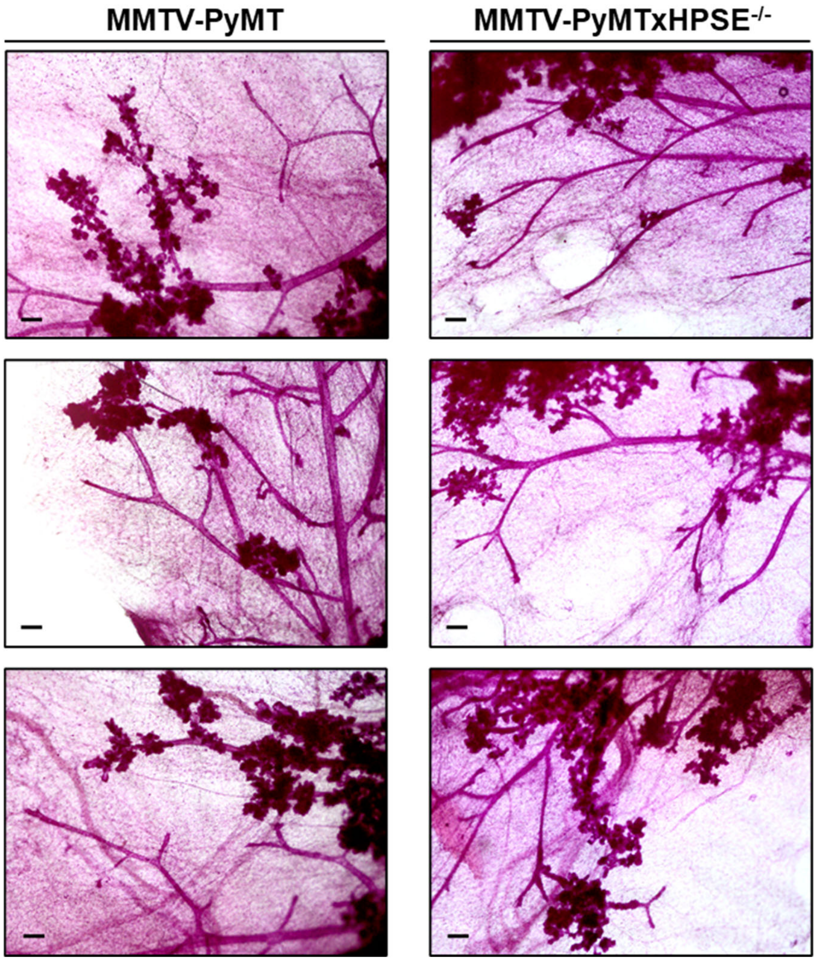

Supplementary Figure S2. Mammary gland architecture of MMTV-PyMT and MMTV-PyMTxHPSE$^{-/-}$ mice at the ethical tumour volume end point

Whole-mounted 4th inguinal mammary glands of MMTV-PyMT and MMTV-PyMTxHPSE$^{-/-}$ mice excised at the ethical tumour volume end point appeared similar in architecture upon visual observation. Representative images of one mammary gland per mouse, *n* = 3 per group. Scale bars = 50 µm.

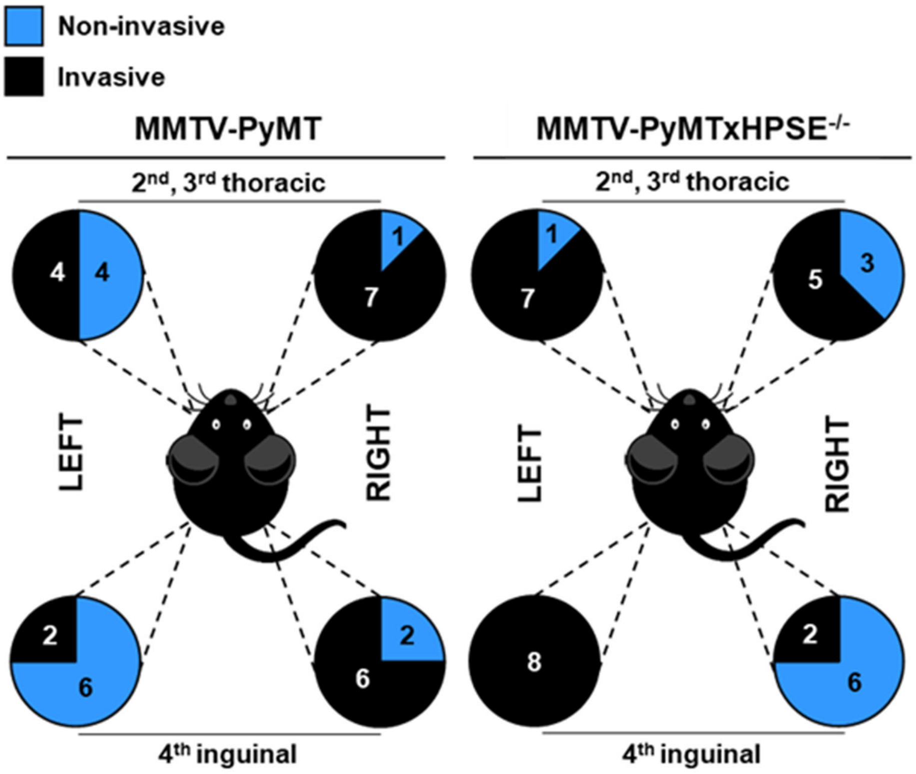

Supplementary Figure S3. The role of HPSE in early mammary tumour development in MMTV-PyMT and MMTV-PyMTxHPSE$^{-/-}$ mice

A pie-chart representation of invasive vs non-invasive phenotypes observed in each of the excised mammary glands of MMTV-PyMT and MMTV-PyMTxHPSE$^{-/-}$ mice as reported in figure 6 (n = 8).

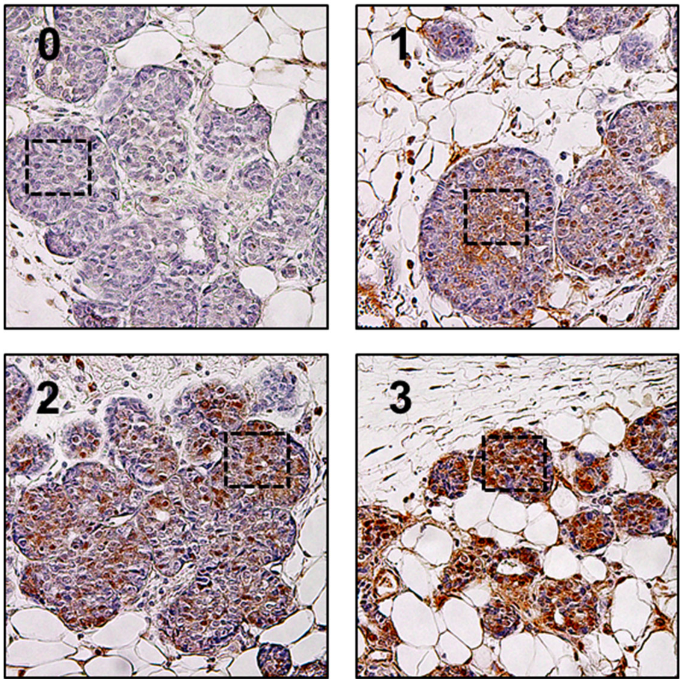

Supplementary Figure S4. H-scoring of mammary tumour sections following IHC staining

A score of 0 – 3 was assigned to four distinct levels of staining, from no staining to highest intensity.

Supplementary Figure S5. Original Western Blots for Figure 1A.

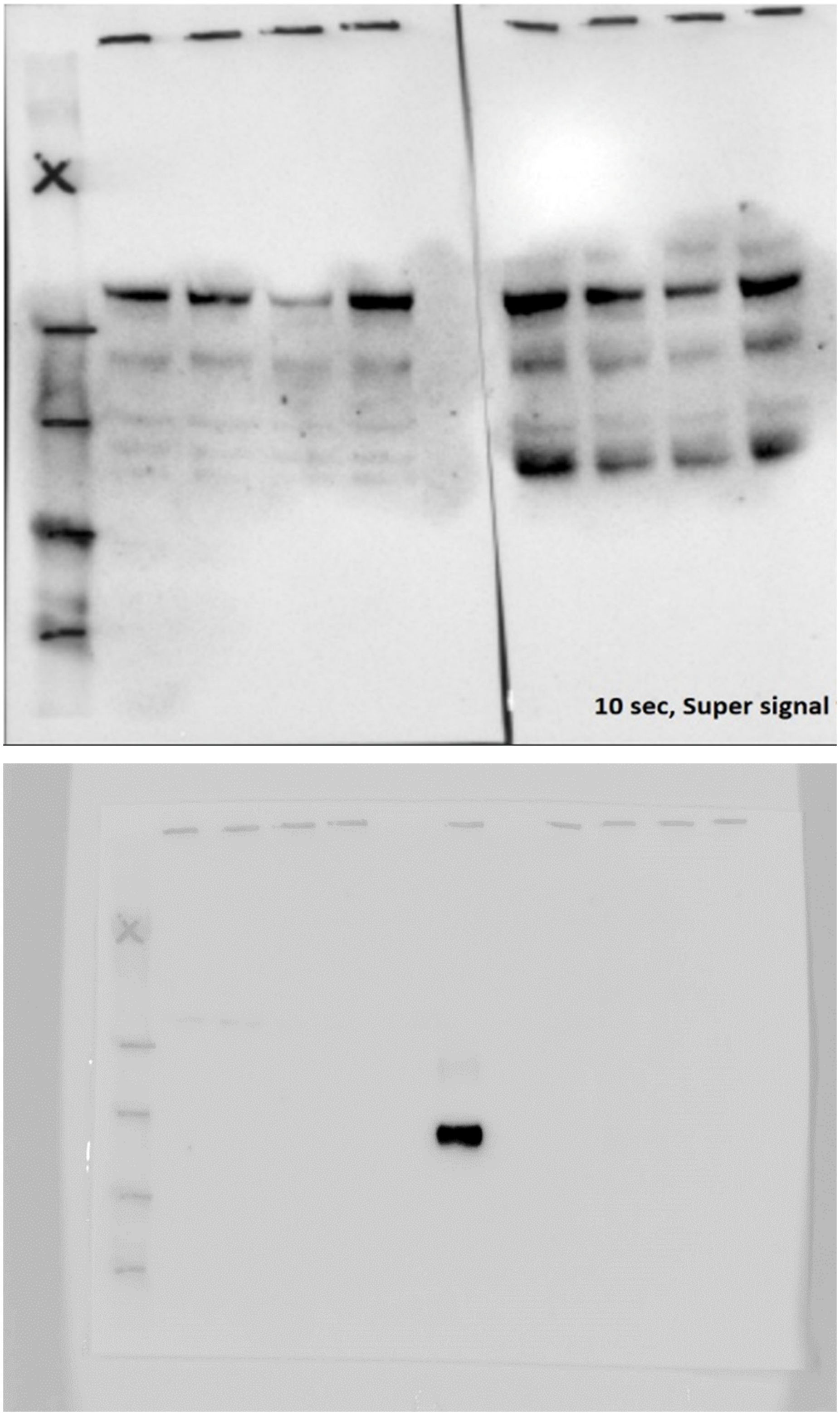

Supplement: Supplementary file 1 [file cancers-15-03062-s001.zip › cancers-2403821-supplementary.pdf]
